# Understanding measurement of postural hypotension: a nationwide survey of general practice in England

**DOI:** 10.3399/BJGP.2025.0025

**Published:** 2025-11-18

**Authors:** Sinéad TJ McDonagh, Rosina Cross, Jane Masoli, Judit Konya, Gary Abel, James P Sheppard, Bethany Jakubowski, Cini Bhanu, Jayne Fordham, Katrina Turner, Sarah E Lamb, Rupert A Payne, Richard J McManus, John L Campbell, Christopher E Clark

**Affiliations:** 1 Exeter Collaboration for Academic Primary Care, Department of Health and Community Sciences, Faculty of Health and Life Sciences, University of Exeter, Exeter, UK; 2 Nuffield Department of Primary Care Health Sciences, University of Oxford, Oxford, UK; 3 London School of Hygiene and Tropical Medicine, London, UK; 4 Primary Care & Population Health Department, University College London, London, UK; 5 Mid Devon Medical Practice, Devon, UK; 6 Centre for Academic Primary Care, University of Bristol, Bristol, UK; 7 Brighton & Sussex Medical School, University of Sussex, Brighton, UK

**Keywords:** blood pressure measurement, frailty, general practice, healthy ageing, hypotension, orthostatic, hypotension, postural, long-term conditions, survey

## Abstract

**Background:**

Postural hypotension is associated with excess mortality, falls, and cognitive decline. Postural hypotension is poorly recorded in routine general practice records. Few studies have explored measurement and diagnosis of postural hypotension in general practice.

**Aim:**

To understand how postural hypotension is measured, diagnosed, and managed in general practice.

**Design and setting:**

This was an online survey of general practice staff in England.

**Method:**

Clinical research networks distributed the survey to practices, seeking individual responses from any clinical staff involved in routine blood pressure (BP) measurement. Responses were analysed according to role and demographic data using descriptive statistics. Multivariable modelling of checking for postural BP measurements was performed.

**Results:**

There were 703 responses from 243 general practices (mean practice-level response rate 17%). Half (362; 51%) of responders were doctors, 196 (28%) nurses, and 77 (11%) healthcare assistants (HCAs). In total, 8% (58/703) did not routinely check for postural hypotension, usually citing time constraints. For the remaining 92%, postural symptoms were the predominant reason for checking (97% responders, 627/645); only 24% cited any other guideline indication for postural hypotension testing. The study found that 77% used sit-to-stand BP measurements; approximately one-quarter measured standing BP for >1 min. On regression modelling, other professionals tested less for postural hypotension than doctors (odds ratios: nurses 0.323, 95% confidence interval [CI] = 0.117 to 0.894, HCAs 0.102, 95% CI = 0.032 to 0.325, and pharmacists 0.099, 95% CI = 0.023 to 0.411).

**Conclusion:**

Awareness of reasons, besides symptoms, and adherence to guidelines for postural hypotension testing, are low. Time is the key barrier to improved testing for postural hypotension. Clarity on pragmatic methods of measuring postural hypotension in general practice would also facilitate measurement uptake.

## How this fits in

Postural hypotension is common in older people and is associated with excess falls, cognitive decline, and mortality. Despite affecting up to 20% of older adults, postural hypotension is rarely recorded in English primary care records (<1%). Few studies have explored postural hypotension measurement and management methods in general practice. This survey of over 700 primary care health professionals showed limited awareness of reasons to check for postural hypotension unless postural symptoms were reported, and low adherence to formal standards of testing; perceived lack of time and lack of awareness were suggested as key barriers to assessment.

## Introduction

Orthostatic or postural hypotension describes the fall in blood pressure (BP) on rising to a standing position from sitting or lying. It is usually defined as a sustained reduction in either systolic BP ≥20 mmHg or diastolic BP ≥10 mmHg within 3 min of standing from lying.^1^ Postural hypotension is common, particularly among adults aged ≥60 years.^2^ A recent systematic review (by this same author group, McDonagh *et al*) found a pooled prevalence of postural hypotension, according to the consensus definition stated, of 19% (95% confidence interval [CI] 15 to 25) for 23 studies of primary care cohorts (median cohort age 73 years; interquartile range [IQR] 63–79 years).^3^ However, in contrast to such data, another study by this same author group (Bhanu *et al*) found a recorded diagnosis of postural hypotension in <1% of 3 million UK primary care records for >50 -year-olds.^4^ This discrepancy suggests that postural hypotension is currently underdiagnosed, and/or underrecorded, in UK primary care.

Such a recording deficit, irrespective of cause, is important. Postural hypotension is a major contributor to falls; it has been prospectively and independently associated with increased risks of falls.^5–8^ In 2013, falls were estimated to cost the NHS £2.3 billion per year (equating to £3.1 billion in 2025).^9^ The prevalence of both falls and postural hypotension rise steeply with age and falls are the leading cause of disability and death from injury for people >75 years of age.^2,3,6,10^ Postural hypotension is also associated with between a 36% and 50% increase in hazards of all-cause mortality,^11–13^ and up to 42% increased risks of cognitive impairment or development of dementia.^14^ Consequently, recognition and diagnosis of postural hypotension is important to mitigate such risks.

Primary care can play a central role in identifying postural hypotension at an early stage; however, current guidance is varied and is based on limited evidence.^15^ About half of people with postural hypotension are asymptomatic, therefore detection of postural hypotension cannot rely on symptoms and should be targeted at those most likely to experience it.^16^ UK and international hypertension guidelines recognise the associations of postural hypotension with older age or comorbidities by advocating checking of standing BP in people >80 years of age, or with diabetes, as well as in those reporting postural symptoms.^17,18^ However, current evidence suggests that primary care practice in testing for postural hypotension is almost exclusively symptom led. The same author group, previously found in a survey of GPs from Southwest England that, without postural symptoms, postural hypotension was not routinely tested for, ostensibly because of workload implications. This, and concerns over the validity of applying lying-to-standing diagnostic thresholds to sit-to-stand measurements, means that many asymptomatic people with postural hypotension go undiagnosed.^19,20^

The reasons underlying the paradox of low recording rates for postural hypotension in primary care records despite much higher expected prevalence of postural hypotension have not been researched in detail: a range of general practice- and population-level factors can have an impact on BP care.^15,19,21,22^ It is important that any general practice intervention is considered both worthwhile and feasible if it is to be successfully implemented.^23^ A thorough understanding of current practices, barriers to, and facilitators of postural hypotension testing, diagnosis, and management is needed to achieve this. This article details findings from a national survey that aimed to describe current practice for postural hypotension measurement in English general practice.

## Method

A survey of general practice clinical staff with responsibility for BP measurements in England was undertaken. Questions were designed to ascertain current practice in the measurement, diagnosis, and management of postural hypotension in English general practices.

### Questionnaire design and distribution

The online survey (Supplementary Information S1) was designed, taking account of relevant national and international guidelines,^15,17,18,24^ and existing research findings,^3,4,15,19,25^ incorporating the clinical experiences of a multidisciplinary research team. The authors also consulted with GP and nursing colleagues, and the project patient and public involvement and engagement team members throughout the design process. The survey was hosted on a secure online survey platform (Joint Information Systems Committee [JISC], Online Surveys v2.0, Bristol, UK) and all data processed in compliance with General Data Protection Regulations.

A draft survey was piloted by healthcare staff in general practices independent of the research team and the survey was finalised in response to their feedback before going live.

### Recruitment and participants

The survey was publicised and distributed by 16 of the 17 regional National Institute for Health and Care Research clinical research networks (CRNs) in England. Recruitment was supported by social media posts and by CRN reminders. General practices in regions with no or few responses received at least three reminders to maximise variation in settings and practice populations. Access to the survey link was restricted to CRN advertising only, to permit estimation of the general practice-level denominator for responses. Consequently, distribution was to research-active general practices engaged with their CRN — representing approximately 60% of all general practices in England.

Responses were sought from any general practice-based primary care staff involved in day-to-day BP measurement (that is, GPs, practice nurses, healthcare assistants [HCAs], pharmacists, paramedics, and other professionals sometimes involved in BP assessment). General practices were invited to distribute the survey invitation to all such staff members.

### Sample size

The authors set out to initially approach 500 general practices. From experience, the authors anticipated a 30% practice-level response rate, that is, around 150 general practices with, on average, 10 multidisciplinary team members regularly measuring BP totalling around 1500 potential responders. A 25% response rate was assumed, resulting in 375 responders. This would result in a 95% CI = 7% to 13% for an observed proportion of 10% (for example, responders routinely measuring standing BP) and 0.3% to 2.7% around an observed proportion of 2%.

### Data management and analysis

Data were exported from the JISC platform as an Excel spreadsheet, anonymised, and then analysed using Stata v18 (Statacorp, Texas, US). As it was anticipated that approaches to postural hypotension vary between members of the multidisciplinary team, the primary unit of analysis was based on individual responses. Survey responses were summarised using descriptive statistics, and between-group differences were tested with χ^2^ or Wilcoxon rank-sum tests as appropriate for the data. Responses were compared across responder age category, gender, professional role, training status, time in practice, as well as practice list size, rurality, and deprivation status of patient populations where ≥10 responses were received per group. As this resulted in 376 cross-comparisons (47 questions across eight responder/practice characteristics) the authors were alert to the potential for chance findings because of multiple testing. To address this risk, the authors initially examined a matrix of overall patterns of significance and focused on those characteristics where associations were significant at *P*<0.05 for ≥20% of responses, and thus highly unlikely to represent chance findings. In addition, the authors restricted reporting of findings to a significance level of *P*<0.001.

Exploration of the data showed substantial variations in reported practice-level categorical characteristics (rurality, practice size, and deprivation status) between individual responses within general practices. Therefore, using practice codes, the authors matched publicly available data (Office for National Statistics 2011 Rural or Urban Classification, quintiles of Office for Health Improvement and Disparities 2019 score, and quintiles of NHS Digital data for practice size November 2022) for use in analyses. Consequently, analyses using matched data were restricted to responses that included a valid practice code.

A mixed-effects logistic regression model was used, with a random intercept for practice, to examine factors associated with checking for postural hypotension in primary care. Covariates included in this model were age category, gender, professional role, training status, time in practice, practice list size, rurality, and deprivation status of patient populations.

## Results

### Description of survey responders

The survey was open from 10 August to 8 December 2022 and distribution to 1551 general practices was confirmed by 10 CRNs in total. There were 703 individual responses; 628 (89%) included some practice identifier for 243 individual general practices (overall practice response rate 17%, individual CRN response rates ranged from 0% to 69%, Supplementary Table S1). All 703 responses were included in descriptive analyses. Practice identifiers were matched to NHS Business Services Authority practice codes for 606 responses from 221 practices; of these, a single response was received from 116 practices (52%) and ≥3 responses from 33% of general practices (maximum 18 responses, Supplementary Table S2 & Figure S1).

Of the 703 responses, half (362, 51%) of the responses received were from GPs, 196 (28%) from nurses, 77 (11%) from HCAs, and 68 (10%) were from people with various other roles. Most responders (631/703, 90%) stated that they were fully qualified; 40 (6%) described themselves as trainees (Table 1).

The median age was 45 years (IQR 38–53), 505/703 (72%) identified as female, 187/703 (27%) male, and one as other; 10 preferred not to say; five identified by a gender different from their birth sex. Proportions of female responders varied from 55% of doctors to 95% of nurses and 97% of HCAs. Responders had worked in general practice for a median of 11 (IQR 5–20) years.

**Table 1.** Roles of survey responders

Professional role	Total^a^
GP	362 (23)
Nurse (any)	196
Practice nurse	111 (1)
Advanced nurse practitioner	31 (4)
Nurse prescriber	19
Nurse practitioner	15
Research nurse	10
Nursing associate	7 (4)
Other nurse^b^	3
HCA	77 (3)
Pharmacist	25
Paramedic	11
Physician associate	6
Advanced clinical practitioner	6 (1)
Other^c^	4
Urgent care practitioner	3
Manager	3
Pharmacy technician	3
Phlebotomist	3 (2)
Research psychologist	3 (2)
Physiotherapist	1
Total	703

^a^Figures in parentheses are the numbers describing themselves as trainees. ^b^Other nurse includes one frailty matron, one older adult nurse, and one respiratory nurse specialist. ^c^Other includes one each of administrator, clinical care coordinator, research practitioner, and 'inactive' (own description) who could not be classified into the above groups. HCA = healthcare assistant.

Most responses (615/703, 87%) were from personnel working in medium- or larger-sized general practices (Supplementary Table S3) reported to be in urban (385/703, 55%) or semi-rural (289/703, 41%) settings (Supplementary Table S4). Responders described their general practice populations as affluent (130/676, 19%), neither affluent nor deprived (319/676, 47%), or deprived (227/676, 34%).

It can be seen from Suppplementary Information S2 that most (72%, 34/47) associations between role and question responses were significant at *P*<0.05. This is much higher than the 2.35 of 47 associations that would be expected to return a *P*-value <0.05 by chance alone, therefore the authors concluded that these associations were broadly likely to be real. Overall, 43% (20/47) of associations with gender were statistically significant, again much higher than expected by chance. Trainee status, time in role, and age had fewer significant associations (17%, 8/47; 23%, 11/47; and 26%, 12/47), but still many more than expected by chance, although some caution is encouraged when interpreting individual associations that will be more susceptible to type 1 errors than the *P*-value indicates. Significant associations with practice-level variables were entirely consistent with chance.

### Measuring postural BP changes

The study found that 58 of 703 (8%) responders did not routinely check for postural BP changes, with variation by role from 10 of 362 (3%) GPs and 16 of 196 (8%) nurses to 14 of 77 (18%) HCAs (Table 2).

Common reasons given for not checking were ‘not part of my role’ (22/58, 38%), ‘I don’t have the time’ (14/58, 24%), or ‘Postural changes have never been mentioned to me before’ (13/58, 22%; Supplementary Table S5).

**Table 2.** Proportions checking for postural changes by role

Professional role^a^	Do you check for postural blood pressure changes?		Total, *n*
	**No, *n* (%)**	**Yes, *n* (%)**	
GP	10 (3)	352 (97)	362
Nurse	16 (8)	180 (92)	196
Healthcare assistant	14 (18)	63 (82)	77
Pharmacist	5 (20)	20 (80)	25
Paramedic	0 (0)	11 (100)	11
Total			671

^a^Where >10 role responses received ; P<0.001 for differences by role. Roles with <10 responses (as summarised in Table 1) are not included.

The dominant reason given by the 645 responders for considering measurement of postural BP changes was suspected postural symptoms (627/645, 97%); all other reasons were cited much less frequently, the commonest of these being checking >80-year-olds cited by 157/645 (24%; Table 3).

GPs were less likely than nurses, HCAs, pharmacists, or paramedics to measure postural BP changes for people with diabetes, hypertension, or within a medication review; however, GPs did more often check for postural hypotension in people with Parkinson’s disease (Supplementary Information S2).

**Table 3.** Reasons for considering measurement of postural BP changes, *N* = 645

Reason	*N*	%
Check when symptoms present^a,b^	627	97
Check when diabetes present^a^	69	11
Check when reviewing hypertension^b^	109	17
Check when Parkinson's disease present^b^	134	21
Check when patients are >80 years^a^	157	24
Check when reviewing medications^a^	77	12
Check if asked by GP^c^	40	6
Check for other specified reason^d^	18	3

^a^Indicated in National Institute for Health and Care Excellence hypertension guidance CG136 (last updated 2023).^17 b^Indicated in European Society for Hypertension 2023 guideline.^18 c^This reason specifically reported by 23 (13%) nurses and 17 (27%) healthcare assistants only. ^d^Other reasons (number) given were: frailty (2), research (6), low BP (4), patient unwell (3), eating disorders (2), and borderline BP control (1).

Over three-quarters of responders (486/629, 77%) used a sit-to-stand technique for postural BP measurement, 141 of 645 (22%) used lying- to-stand, and two measured lying to sitting BP changes. Nurses and HCAs used a lying-to-stand protocol more often than GPs, pharmacists, or paramedics (Supplementary Table S6).

**Figure 1.**
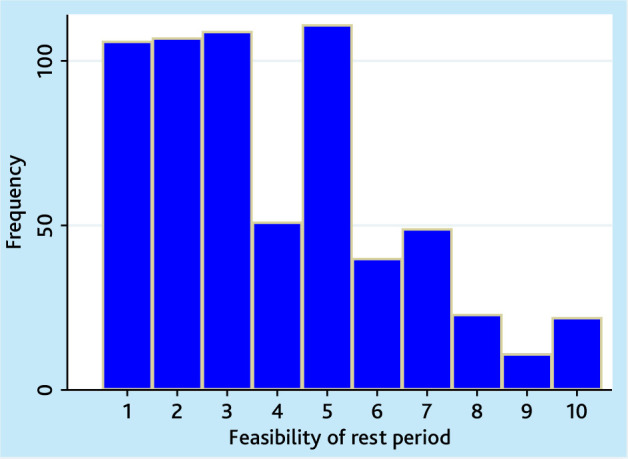
Feasibility of including a rest period before measurement. Visual scale 1–10: 1 not feasible at all to 10 completely feasible.

Only two-thirds (401/629; 64%) used a sitting rest period before standing; the median feasibility rating (out of 10) of using a rest period was 3 (IQR 2–5; Figure 1). GPs were less likely to do this than other team members (Supplementary Table S7) and rated feasibility of doing so lower than any other health professionals. The median rest period was 5 (IQR 2–5) min. A median of 2 (IQR 1–3) resting measurements were reported, with doctors collecting fewer than nurses or healthcare assistants (Supplementary Table S8).

**Figure 2.**
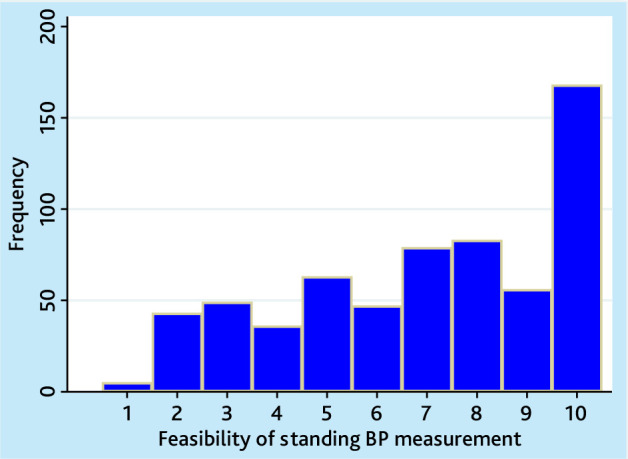
Feasibility of obtaining a standing blood pressure (BP) measurement. Visual scale 1–10: 1 not feasible at all to 10 completely feasible.

Obtaining standing BP measurements was rated more feasible than using a rest period (median rating 7; IQR 5–10; Figure 2).

Half of responders (315/629; 50%) measured standing BP once (median 1, IQR 1–2); GPs were least likely to repeat standing BP measurements more than once (Supplementary Table S9). Two-thirds (420/629, 67%) of responders measured postural hypotension less than a minute after standing (Supplementary Table S10).

### Diagnosis of postural hypotension

There was some evidence that the adoption of set diagnostic criteria reduced with increasing age and/or years in practice (Supplementary Information S2). The consensus diagnostic thresholds for postural hypotension proposed by Freeman *et al* (≥20/10 mmHg) were most frequently cited (by 346/629, 55%), whereas 48 of 622 (8%) cited the lower sit-to-stand diagnostic threshold of ≥15/7 mmHg proposed by Shaw *et al* (Table 4).^1,20^

There was no evidence of an association between resting posture (that is, lying or sitting) and choice of diagnostic criterion (Supplementary Table S11). A majority of nurses (98/170; 58%) and HCAs (47/58; 81%) did not diagnose postural hypotension, instead passing results to the GP for diagnosis; consequently, approaches to diagnosis differed markedly according to role (Supplementary Table S12).

**Table 4.** Diagnostic criteria adopted for diagnosing postural hypotension, total of 629 responses

Criterion	*N*	%
A drop in systolic blood pressure by at least 15 mmHg or a drop in diastolic blood pressure by at least 7 mmHg within 3 min of standing (Shaw)	48	8
A drop in systolic blood pressure by at least 20 mmHg or a drop in diastolic blood pressure by at least 10 mmHg within 3 min of standing (Freeman)	346	55
I check for postural changes in blood pressure but I don’t diagnose	155	25
I don’t work to a particular diagnostic definition	73	12
I don’t know	2	0.3
Other^a^	5	0.8

^a^Other, one each of: a drop of 10 mmHg, a drop of 15/10 mmHg, a drop consistently >20 mmHg; a drop of 20/20 mmHg after 1 minute, and a drop of 10–20 mmHg systolic.

### Recognition and management of postural BP changes

Most responders had observed postural BP falls (595/629, 95%). Their usual response was to review BP-lowering medication (401/595, 67%); 96 (16%) would standardise their care to standing BP values (Supplementary Table S13). Considering postural rises in BP, 480 of 629 (76%) reported awareness of BP rising with postural changes, with considerable heterogeneity of actions taken in response. The commonest choice (195/480, 41%), particularly for GPs, was to take no action (Supplementary Table S14). Continuing to monitor changes was an option chosen across all professional roles.

Although the COVID-19 pandemic had minimal impact on postural BP measurement, with only 46 of 703 (7%) GPs and nurses changing their approach, more GPs (208/703, 30%) did change their approach to BP measurement in general, mainly by increasing use of patients’ home BP measurements (Supplementary Information S3 and Supplementary Tables S15 & S16).

### Home postural BP measurement

Almost half (335/703, 48%) of responders had asked patients to collect home postural BP measurements. Another quarter (178/703, 25%) had never considered this approach and 80 of 703 (11%) thought this might be unsafe for patients (Supplementary Table S17). Safety was an issue of concern for trainees more often than for fully qualified staff (12/40, 30% versus 68/663, 10%). GPs considered patients unable to undertake home postural testing (82/362, 23%) more often than nurses or HCAs (23/196, 12% and 0/77, 0%, respectively); trainees also made this judgement twice as often as qualified staff (13/40, 33% versus 99/663, 15%). Responders relied on sphygmomanometers belonging to the patient in two-thirds of cases (234/335, 70%); the usual alternative was a practice-owned machine on loan (Supplementary Table S18).

Sphygmomanometer types used in general practice were evenly split between automated (360/680, 53%) or manual (318/680, 47%) devices (Supplementary Table S19).

### Modelling of measurement of postural hypotension

Full data for 561 responders contributed to regression modelling. Professional role was strongly associated with practitioner decisions to test for postural hypotension. When compared with GPs, non-GP staff were far less likely to test (odds ratios [ORs] for nurses 0.323 [95% CI = 0.117 to 0.894], HCAs 0.102 [95% CI = 0.032 to 0.325], pharmacists 0.099 [95% CI = 0.023 to 0.411]; overall *P*<0.001).

There was no evidence of an association with any other factor considered after adjustment for the other factors had taken place, except for practice deprivation; professionals working in practices serving populations with higher deprivation being increasingly likely to test (OR for most deprived versus least deprived quintile 3.942 [95% CI = 1.015 to 15.307]; overall *P* = 0.095; Table 5).

**Table 5.** Multivariable model of checking for postural hypotension

Variable	Odds ratio	95% confidence interval	*P*-value
Role			
GP (ref)	1	NA	
Nurse	0.323	0.117 to 0.894	
HCA	0.102	0.032 to 0.325	
Pharmacist	0.099	0.023 to 0.411	<0.001
Status			
Fully qualified (ref)	1	NA	
Trainee	0.871	0.146 to 5.191	0.879
Gender			
Male (ref)	1	NA	
Female	0.846	0.422 to 1.699	0.639
Age quintile, years			
20 to 36 (ref)	1	NA	
37 to 42	0.798	0.209 to 3.045	
43 to 49	0.681	0.182 to 2.540	
50 to 55	0.712	0.174 to 2.913	
56 to 67	0.475	0.114 to 1.987	0.879
Years in practice quintile			
0 to 4 (ref)	1	NA	
5 to 8	1.190	0.368 to 3.842	
9 to 14	0.689	0.222 to 2.138	
15 to 21	1.012	0.264 to 3.874	
22 to 40	1.502	0.324 to 6.967	0.762
Location			
Rural practice (ref)	1	NA	
Urban practice	1.690	0.571 to 4.999	0.343
IMD deprivation quintile			
1 (least deprived; ref)	1	NA	
2	1.183	0.435 to 3.220	
3	2.555	0.822 to 7.943	
4	3.751	1.051 to 13.386	
5 (most deprived)	3.942	1.015 to 15.307	0.095
Practice list size quintile			
2789 to 7724 (ref)	1	NA	
7779 to 10 256	1.023	0.346 to 3.029	
10 293 to 14 902	2.327	0.667 to 8.112	
15 058 to 21 535	1.354	0.421 to 4.349	
21 902 to 67 402	1.564	0.467 to 5.236	0.687
Constant	15.098	1.294 to 176.116	0.030

CI = confidence interval. HCA = healthcare assistant. IMD = Index of Multiple Deprivation. ref = reference category.

## Discussion

### Summary

This national survey examined current practice in the measurement and management of postural hypotension in English primary care. Half of responses were from GPs, the remainder from nurses and allied health professionals. Most (645/703, 92%) responders actively test for postural hypotension, but usually only in response to postural symptoms being reported. Other guideline-recommended reasons for checking, such as age (>80 years) or monitoring of comorbidities such as diabetes or hypertension were reported less often.

Three-quarters of responders used sitting-to-standing measurement of postural hypotension, lying-to-standing being more often used by nurses or HCAs. Overall, only 56% (346/629) were aware that the consensus definition of postural hypotension relies on lying-to-standing measurement.^1^ Use of recommended rest periods before BP measurement, and repetition of resting and standing measurements, were inconsistent.

### Strengths and limitations

The sample of over 700 survey responses was near double that anticipated in planning this survey, making it the largest such survey, to the authors’ knowledge, to date. The recruitment of individuals through practice invitation was pragmatic; it permitted estimation of an overall practice-level response rate of 17.3% (269/1551). General practice identifiers were missing from 10.7% (75/703) of responses, thus the number of individual contributing practices reported is likely to be an underestimate. As the survey was a CRN portfolio study, it was distributed to research-active practices who were incentivised to participate by CRN adoption. The methods did not allow knowledge of individual-level response rates either overall or by profession, so caution may be required in generalising the findings to non-research-active practices or specific clinical groups such as physician associates who were poorly represented. The study did not enquire about the responders’ ethnicity, therefore the level of inclusion for groups of professionals who may be underrepresented in research is unknown. The authors sought recruitment by all 17 English CRNs but it was not possible to elicit the reasons for no responses from some CRNs; one CRN did not distribute the authors' invitations. Despite this, the authors believe that the sample is reasonably representative of English general practices.

Owing to heterogeneity of reporting for practice list size, rurality, and deprivation status between multiple responders within general practices, the study instead used national practice-level data in analyses for consistency. To avoid selective reporting, and to account for the potential impact of multiple testing, the authors summarised all outcomes but only present findings with a significance level of *P*<0.001.

### Comparison with existing literature

The survey referred to relevant national and international guidelines for who should be tested for postural hypotension.^15,17,24^ The same author group has previously found that the prevalence of postural hypotension in older adults approaches 20%, yet a recorded diagnosis of postural hypotension appears in only 1% of English routine primary care records.^3,4^ This study confirms earlier findings that postural hypotension is not currently systematically tested for, rather being led by symptomatic presentation.^15,19^ The low implementation of testing, irrespective of symptoms, for groups of people identified to be at risk of postural hypotension by guidelines may, in part, account for the discrepancy between observed and recorded prevalences of postural hypotension in primary care.

The consensus definition of postural hypotension (≥20/10 mmHg drop) relies on measurement of BP on standing from lying supine (or on tilt-table testing) and was proposed by specialists in neurology and autonomic dysfunction.^1^ BP changes on sit-to-stand testing — the standard of care in this study — differ in magnitude and underdiagnose postural hypotension in comparison with lying-to-standing tests.^26,27^ Consequently, a lower diagnostic threshold has been proposed for sit-to-stand testing (≥15/7 mmHg drop).^20^ Although it seems likely that sit-to-stand testing also underdiagnoses postural hypotension in primary care, all available evidence is from specialised cohorts and settings, making it hard to generalise to the primary care setting. The survey revealed inconsistency in approaches to postural hypotension testing that are likely to also contribute to underdiagnosis of postural hypotension.^28^

### Implications for research and practice

The study findings imply that a minority of GPs do not measure postural changes in BP. However, nurses and HCAs reported performing postural BP measurement at the request of a GP, so it is plausible that GPs may, in fact, simply be delegating measurement to other team members.

Awareness of both the diagnostic criteria for postural hypotension, and for whom testing is recommended, seems suboptimal from the survey findings. As half of all people with postural hypotension either lack, or are unaware of, postural symptoms, systematic testing for postural hypotension cannot be led by patients’ postural symptoms. Healthcare professionals generally recognise dizziness; however, other symptoms such as coat hanger syndrome, platypnoea, blurry or dimmed vision, or nausea can be vague and underrecognised by both patients and professionals.^15,29,30^ Many guidelines advise postural BP measurement protocols based on the consensus definition but differ from each other (in terms of rest periods, standing periods, and numbers of required measurements).^3,15^ These have not been developed or validated in primary care populations; this study found diagnostic confusion around postural hypotension protocols, suggesting that clarity on methods of practical primary care measurement and diagnosis of postural hypotension is needed.

Diagnosis of postural hypotension is a prerequisite of any intervention. Fall prevention interventions are highly cost-effective, but require recognition of risk markers such as postural hypotension to be implemented successfully.^9,24,31^ Management of postural hypotension *per se* targets symptom control, mainly through non-pharmacological interventions and medication review. Evidence for the impact of interventions for postural hypotension on long-term outcomes such as mortality is lacking, and the effects of ameliorating postural hypotension symptoms at the expense of BP elevation (by either medication withdrawal or prescribing of pressor agents) on long-term outcomes is unknown.^32^

In November 2023, taking account of the authors’ current findings, the National Institute for Health and Care Excellence (NICE) amended the guidance on postural hypotension testing.^17^ They accepted sit-to-standing testing as a pragmatic means of initial assessment. However, if the sit-to-standing test is not diagnostic, formal lying-to-standing measurement should be used where suspicion of postural changes remains.^17^ The current study's findings have confirmed that time and practical considerations are a significant barrier to uptake of postural hypotension measurement, raising concerns that this modified NICE guidance may not be fully feasible in primary care.

Although home BP measurement is used routinely to manage hypertension, it was only occasionally considered for diagnosis of postural hypotension with some responders expressing concerns over safety. There is limited evidence to support this process at present. It requires further research but appears to be safe and has the potential to increase incident diagnosis of postural hypotension.^33^

In conclusion, this survey found that postural hypotension is not routinely tested for in English primary care in the absence of postural symptoms. Testing, when it does occur, does not follow diagnostic criteria and standards, and there is little awareness of current guidelines, the prevalence of asymptomatic postural hypotension, and its associated conditions (for example, diabetes). Practice considerations, diagnostic confusion, and the absence of primary care evidence are significant barriers to greater uptake of postural hypotension testing.

## Data

The authors do not have consent to openly share the data but inquiries to the corresponding author are welcomed.
